# Ennore, Boscombe

**Published:** 1903-06-20

**Authors:** 


### ENNORE, BOSCOMBE.

### AN ESTABLISHMENT FOR THE TREATMENT OF DIABETES.

It has often been a matter for surprise and regret to us that institutions for the proper treatment of diabetes and allied diseases have never been organised in England, and we welcome the appearance of the first one. It will be no longer necessary for the invalid to incur the expense and fatigue of a long journey to Karlsbad or some other "cure" resort. And although we may admit that the climate and general surroundings of Karlsbad and kindred places have something to do with the admirable results often obtained thereat, it is unquestionable that the treatment of diabetes is essentially one of strict attention to diet and hygienic arrangements. For any single member of a family to rigidly adhere to these in his own home is always difficult and often quite impossible. Hence the boon which this establishment at Bournemouth will confer to sufferers.

Bournemouth can be reached from Waterloo in a little over two hours, and the house is not more than 15 minutes' drive from the station. It is a fine modern mansion, having about 20 bedrooms, and of that number only two face the north, so well has the mansion been planned. The aspect is south and south-west. It stands high, and splendid views of the sea and the coast-line can be obtained. It is close to Boscombe Pier and the pretty public gardens near the shore, where regulated exercise can be secured by the patients, and almost every form of amusement can be had in the immediate neighbourhood. The mansion itself has a fine billiard-room, and both the public rooms and bedrooms are above the average of institutions, not only as regards size but in comfort and furnishing. There is a well fitted up laboratory, and here the physician-superintendent, Major Grant, formerly of the Indian Medical Service, carries out the clinical examinations and analyses. It is not always easy to make diabetic food attractive to patients; but we can testify that Major Grant has solved the problem, and no one need feel the least misgivings on the score of the cookery. In this particular disease Major Grant has had unusual experience, and records of his career show that he was for ten years Physician to the Madras Hospital, and was also lecturer on Clinical Medicine. He is author of a work on Hygiene, and was at one time Professor in that Department.

One great advantage which this establishment has over Karlsbad is that it is open all the year round, and patients can choose their own time for treatment instead of being limited to the four or five months during which the foreign places are accessible. Add to this fact the home comforts which surround the invalid at Ennore, and there can be no doubt as to which is preferable. The inclusive rate is £5 5s. a week, and this includes all medical charges save the first consultation.

We have said that this pioneer must be a boon to many sufferers, and it will also free the practitioner, who may send his patient there, from many misgivings as to whether his patient is having properly regulated diet in his own home, the absence of which often causes the practitioner much disappointment.

